# Liquid Biopsy from Bile-Circulating Tumor DNA in Patients with Biliary Tract Cancer

**DOI:** 10.3390/cancers13184581

**Published:** 2021-09-12

**Authors:** Jin-Yi Han, Keun Soo Ahn, Tae-Seok Kim, Yong Hoon Kim, Kwang Bum Cho, Dong Woo Shin, Won-Ki Baek, Seong-Il Suh, Byeong-Churl Jang, Koo Jeong Kang

**Affiliations:** 1 Department of Surgery, Division of Hepatobiliary and Pancreatic Surgery, Keimyung University Dongsan Hospital, Daegu 1035, Korea; esr0319@naver.com (J.-Y.H.); gskim80094@dsmc.or.kr (T.-S.K.); hbps@dsmc.or.kr (Y.H.K.); kjkang@dsmc.or.kr (K.J.K.); 2 Department of Internal Medicine, Division of Gastroenterology, Keimyung University Dongsan Hospital, Daegu 1035, Korea; chokb@dsmc.or.kr (K.B.C.); delight0618@dsmc.or.kr (D.W.S.); 3 Department of Microbiology, Keimyung University School of Medicine, Daegu 1095, Korea; wonki@dsmc.or.kr (W.-K.B.); seongil@dsmc.or.kr (S.-I.S.); 4 Department of Molecular Medicine, Keimyung University School of Medicine, Daegu 1095, Korea; jangbc123@gw.kmu.ac.kr

**Keywords:** circulating tumor DNA (ctDNA), *KRAS*, biliary tract cancer (BTC), droplet digital polymerase chain reaction (ddPCR), transcriptomic sequencing

## Abstract

**Simple Summary:**

Utilization of cell free DNA for diagnosing and monitoring patients with biliary tract cancers is emerging and promising. The strength of the present study is in its description of a novel approach using bile rather than blood or tissue samples, which is particularly relevant in biliary tract cancers. This paper largely serves as a proof of concept that ctDNA from bile is potentially feasible.

**Abstract:**

Although liquid biopsy of blood is useful for cancer diagnosis and prediction of prognosis, diagnostic and prognostic value of ctDNA in bile fluid for BTCs are not clear yet. To determine whether liquid biopsy for circulating tumor DNA (ctDNA) can replace tissue biopsy when assessing somatic mutations in biliary tract cancers (BTCs). Bile samples were obtained from 42 patients with BTC. Matched formalin-fixed paraffin-embedded (FFPE) samples were obtained from 20 of these patients and matched plasma samples from 16 of them. Droplet digital PCR (ddPCR) was used for detection *KRAS* somatic mutation. *KRAS* mutations were identified in the bile ctDNA of 20 of 42 (48%) patients. Patients with mutant *KRAS* showed significantly worse survival than those with wild-type *KRAS* (2-year survival rates: 0% vs. 55.5%, respectively; *p* = 0.018). There was 80.0% mutational concordance between the paired bile ctDNA and FFPE samples, and 42.9% between the plasma and FFPE samples. On transcriptomic sequencing of one set of paired bile and FFPE samples, expression level of *KRAS*-associated signaling oncogenes in the bile and tissue samples showed a strong positive correlation (*r =* 0.991, *p* < 0.001). Liquid biopsy of bile reliably detect mutational variants within the bile ctDNA of BTC patients. These results suggest that bile is an effective biopsy fluid for ctDNA analysis.

## 1. Introduction

Biliary tract cancers (BTCs), which include gallbladder cancer (GBC), ampullary carcinoma (AC), and extrahepatic cholangiocarcinoma (eCCA), are rapidly growing tumors that are usually lethal [1,2,3]. The 5-year survival rate for patients with advanced or metastatic BTC is <10% [4,5,6]. A tissue biopsy is needed for diagnosis of BTCs, but it cannot be routinely performed due to its invasiveness. To overcome this limitation, less-invasive techniques for determining tumor heterogeneity and the molecular changes in cancer cells are required [7].

Liquid biopsy can be useful for cancer detection, monitoring, and management. Fragmented DNA circulates in the cell-free component of whole fluids. The cell-free circulating tumor DNA (ctDNA) in the blood is DNA released from apoptotic, circulating, or living tumor cells. ctDNA is about 140 nucleotides long and has a half-life of about 1.5 h. The analysis of ctDNA provides a noninvasive way to assess the genetic profiles of cancers in real time [8]. In healthy individuals, the concentrations of circulating cell-free DNA (cfDNA) in the blood tend to range between 1 and 10 ng/mL plasma [9,10]. Recently, noninvasive ctDNA genotyping of plasma has become a cost-effective alternative to tissue biopsies in the diagnosis and management of many cancers [11]. The potential role of ctDNA in the treatment of BTCs is especially important because endoscopic or percutaneous biopsy is invasive and its accuracy is poor [12]. However, the diagnostic accuracy of ctDNA in blood and the concordance of the results obtained with blood and tissue samples vary in many studies, so the diagnostic value of blood ctDNA for BTCs is still limited [13,14]. In cases of BTC, bile fluid directly contacts the tumor cells, and tumor-derived materials may be abundant in bile. Therefore, bile could be the ideal biofluid for the exploring biomarker and molecular analysis of BTC [15,16].

Recent molecular analyses have identified several markers of poor prognosis and the activation of oncogenic pathways. *KRAS* is frequently mutated in largely diffuse tumors, such as pancreatic cancers, colon cancers, and BTCs [1,4,17,18]. The frequency of *KRAS* mutations in BTCs is 9–40% [19], and *KRAS* mutation has been associated with perineural invasion, advanced stage, and poor prognosis in these patients [20]. These mutations occur and cluster in several hotspots in the gene, and codons 12 and 13 are most frequently affected [21]. The four most frequent KRAS mutations (G12D, G12V, G13D, and G12C) account for 83% of all KRAS mutations. The *KRAS* mutation in codon 12 is especially associated with worse survival in patients with eCCA [4], and the presence of *KRAS* mutations is associated with resistance to several targeted treatments, including tyrosine kinase inhibitor and anti-epidermal growth factor antibody treatments [22,23]. Therefore, the detection of *KRAS* mutations may be important in targeted approaches to treatment. Considering the invasiveness and low accuracy of tissue biopsy in BTCs [12], liquid biopsy, including the detection of *KRAS* changes in ctDNA, may be valuable.

Few studies have assessed the diagnostic and prognostic value of ctDNA in bile fluid for BTCs. Because *KRAS* mutations are frequent and have prognostic implications, we focused on the detection of these mutations in bile fluid. In this study, we used droplet digital PCR (ddPCR) to detect somatic mutations in *KRAS* in liquid biopsy samples to assess whether this technique can replace tissue biopsy as a diagnostic and prognostic tool for BTCs. For that, we compared the *KRAS* mutations detected in tissue, plasma, and bile samples. In addition, expression of *KRAS*-associated signaling oncogenes with next-generation sequencing (NGS), and analyzed correlations of expression among bile, and paired tissue and plasma.

## 2. Materials and Methods

### 2.1. Patients

This prospective study was approved by the Institutional Review Board of Keimyung University Dongsan Medical Center (DSMC 2018-06-054) and included the secondary use of human-derived materials. We obtained bile fluids from 46 individuals, including 42 patients with BTCs (GBC, AC, eCCA), and 4 patients with benign biliary tract disease (CBD stones) as a control, at Keimyung University Dongsan Hospital between August 2018 and March 2020. The diagnosis of BTC was based on a tissue sample obtained by surgical resection, needle biopsy or cytology with endoscopic retrograde cholangio pancreatography (ERCP) or percutaneous transhepatic biliary drainage (PTBD). When eCCA patients had isolated CCA without metastatic lesions and their general status was appropriate for surgery, curative surgical resection was performed. However, when patients had metastatic lesions or refused surgical resection, we administered chemoradiotherapy or palliative therapy [24]. The tumor stage was classified according to the 7th edition of the American Joint Committee on Cancer (AJCC) classification of malignant tumors. The AJCC stage was assessed based on clinical (in patients treated with chemotherapy or radiotherapy) or pathological data (in patients treated with curative surgical resection). Bile samples from both groups were collected at the time as PTBD to relieve cholangitis or obstructive jaundice (for the patients with BTCs) or to relieve cholangitis and remove the CBD stones (for the control). In both groups, the bile fluid was collected after cholangitis was resolved, and at least 3 days after PTBD. Among the 42 patients with BTCs, FFPE samples were available for 20 patients. In 16 patients, plasma (270 μL) was used preoperatively and was stored at −80 °C.

### 2.2. Bile Collection

The collected bile fluid was centrifuged immediately at 16,000× *g* for 10 min at 4 °C to obtain the supernatant (bile). Each pellet was resuspended in chilled phosphate-buffered saline (2-fold), and then centrifuged again 16,000× *g* for 5 min at 4 °C to obtain the bile pellet, as described in the protocol of Abi Zabron (Imperial College, London, England) [24]. All samples were centrifuged with an Avanti J-25I centrifuge (Beckman, CA, USA), and the bile and pellet were stored at −80 °C. After separation, we used these samples in all experiments.

### 2.3. Chemicals and Reagents

The NucleoSpin^®^ cfDNA XS, NucleoSpin^®^ FFPE DNA, and NucleoSpin^®^ Tissue XS kits were purchased from Macherey-Nagel (GmbH & Co. KG, Herborn, Germany). The ddPCR™ *KRAS* Screening Multiplex Kit and ddPCR™ *KRAS* G12/G13 Screening Kit were purchased from Bio-Rad Laboratories (Hercules, CA, USA). Ethanol and all other chemicals were obtained from Sigma (St. Louis, MO, USA).

### 2.4. Preparation of Blood Collection

Blood from patients was collected preoperatively by vacutainer tubes containing heparin as an anticoagulant and was centrifuged 25,000× *g* for 15 min at 4 °C to obtain plasma. Plasma was stored at −80 °C (Keimyung University Dongsan Hospital Biobank).

### 2.5. DNA Extraction from Bile, Plasma, and FFPE Samples

To separate the bile for ctDNA analysis, the collected bile fluid was centrifuged immediately at 16,000× *g* for 10 min at 4 °C to obtain the supernatant (bile), as described in the protocol by Abi Zabron (Imperial College, London, England) [25]. All of the samples were centrifuged with an Avanti J-25I centrifuge (Beckman, Brea, CA, USA), and the bile was stored at −80 °C. The ctDNA was extracted from aliquots of bile (270 ul) with the NucleoSpin^®^ cfDNA XS Kit, according to the manufacturer’s instructions. The DNA was analyzed on an Agilent 2100 Bioanalyzer.

FFPE samples were obtained from 20 patients who underwent surgical resection or endoscopic biopsy. Histological examinations confirmed that these samples contained at least 30% tumor cells. We extracted the DNA from sections (5 mm thick) of the FFPE samples (15 mg) with the NucleoSpin^®^ FFPE DNA Kit, according to the manufacturer’s instructions. The DNA was stored at −20 °C before analysis.

### 2.6. Droplet Digital PCR

DNA fragmentation enables optimal accuracy by separating tandem gene copies, reducing sample viscosity, and improving accessibility. ddPCR was performed on DNA libraries with the ddPCR™ *KRAS* Screening Multiplex Kit and ddPCR™ *KRAS* G12/G13 Screening Kit (Bio-Rad Laboratories) on the Bio-Rad QX200 ddPCR system. A two-dimensional (2D) fluorescence amplitude plot generated with the Quanta Soft™ software showed the wells of mixed mutant and wild-type samples. 2D plots were constructed from the results of the ddPCR *KRAS* multiplex assays applied to the ctDNA from the bile samples from the no-template control (NTC), the BTC patients, and the control patient. The black cluster on the plot represents the negative droplets; the green cluster represents the droplets that were positive for wild-type DNA only; the blue cluster represents the droplets that were positive for mutant DNA only; and the orange cluster represents the droplets that were positive for both mutant and wild-type DNA (Figure 2D–F). The amplification products were detected with a C1000 Tough Thermal Cycler (Bio-Rad Laboratories). The reactions were initiated by incubation at 95 °C for 10 min, followed by 40 cycles of 94 °C for 30 s, 55 °C for 60 s and 98 °C for 10 min. The concentration of DNA was calculated from copy number variations (CNV, copies/mL). The fragment size distributions of the bile ctDNA and FFPE DNA were analyzed with an Agilent 2100 Bioanalyzer (Agilent Technologies, Inc., Santa Clara, CA, USA). The fragment size of the bile ctDNA and FFPE DNA were analyzed with an Agilent 2100 Bioanalyzer using a high sensitivity DNA kit (Bio-Rad Lab).

### 2.7. Direct Extraction of Single-Stranded RNA

In one patient (CA34), for whom bile, tissue, and plasma samples were available, an mRNA-Seq data analysis was performed with NSG to directly compare the gene expression profiles detected in the three biological samples. The total RNA concentrations were calculated with a Quant-iT™ RiboGreen™ RNA Assay Kit (Invitrogen, Waltham, MA, USA). To determine the DV200 value (% of RNA fragments >200 bp), the samples were run on a TapeStation RNA Screen Tape system (Agilent Technologies, Inc.) The total RNA (100 ng) was used to construct sequencing libraries with a SureSelectXT RNA Direct system (Agilent Technologies, Inc.), according to the manufacturer’s protocol. Briefly, total RNA was first fragmented into small pieces with divalent cations at elevated temperatures. The cleaved RNA fragments were copied into first-strand cDNA with reverse transcriptase and random primers, followed by second-strand cDNA synthesis. These cDNA fragments underwent an end repair process, with the addition of a single ‘A’ base, and were then ligated to adapters. The products were purified and enriched with PCR to create the cDNA libraries. To capture the human exonic regions, we used an Agilent SureSelectXT Human All Exon V6 + UTRs Kit (Agilent Technologies, Inc.), according to the standard SureSelect Target Enrichment protocol (Agilent Technologies, Inc.). The cDNA library (250 ng) was mixed with hybridization buffer, blocking mix, RNase blocker, and 5 µL of the SureSelect XT Human All Exon V6 + UTRs capture library. Hybridization to the capture baits was performed at 65 °C with the heated thermal cycler lid option at 105 °C for 24 h on a PCR thermal cycler. The captured library was then washed and subjected to a second round of PCR amplification. The final purified product was quantified with quantitative PCR (qPCR), according to the qPCR Quantification Protocol Guide (KAPA Library Quantification Standards Kits for Illumina^®^ sequencing platforms), and its quality was confirmed with the TapeStation DNA Screen Tape D1000 (Agilent Technologies, Inc.). The indexed libraries were then submitted to Illumina NovaSeq (Illumina, Inc., San Diego, CA, USA), and paired-end (2 × 100 bp) sequencing was performed by Macrogen Incorporated (Seoul, Korea).

### 2.8. SMARTer smRNA Library

The RNA isolated from each sample was used to construct a sequencing library with a SMARTer smRNA-Seq Kit (Illumina Inc.), according to the manufacturer’s protocol. Briefly, the input RNA was first polyadenylated to provide a priming sequence for an oligo (dT) primer. cDNA synthesis was primed with the 3′ smRNA dT Primer, which incorporates an adapter sequence at the 5′ end of each RNA template, to which it adds nontemplated nucleotides. These nucleotides were bound by the SMART smRNA Oligo, enhanced with the locked nucleic acid (LNA) technology for greater sensitivity. In the template-switching step, PrimeScript reverse transcriptase (RT) used the SMART smRNA Oligo as the template for the addition of a second adapter sequence to the 3′ end of each first-strand cDNA molecule. In the next step, full-length Illumina adapters (including the index sequences for sample multiplexing) were added during PCR amplification. The forward PCR primer bound to the sequence added by the SMART smRNA Oligo, whereas the reverse PCR primer bound to the sequence added by the 3′ smRNA dT Primer. The resulting cDNA molecules in the library included the sequences required for clustering on an Illumina flow cell. The libraries were gel-purified with BluePippin, and validated by checking their sizes, purity, and concentrations on an Agilent Bioanalyzer. The libraries were pooled in equimolar amounts, and sequenced on an Illumina HiSeq 2500 instrument to generate 51-base reads. Image decomposition and the calculation of quality values were performed with the modules of the Illumina pipeline.

### 2.9. mRNA-Seq Data Analysis

We preprocessed the raw reads from the sequencer to remove low-quality and adapter sequences before their analysis and aligned the processed reads to the Homo Sapiens genome assembly (GRCh38) using HISAT v2.1.0 [26]. HISAT utilizes two types of indices for alignment (a global, whole-genome index and tens of thousands of small local indices). These two types of indices were constructed with the same Burrows–Wheeler transform (BWT) and graph FM index as Bowtie2. Because it uses these efficient data structures and algorithms, HISAT generates spliced alignments several times faster than Bowtie, and BWA is widely used. The reference genome sequence of Homo Sapiens (GRCh38) and its annotation data were downloaded from NCBI. An assembly of known transcripts was then processed with StringTie v2.1.3b [27,28]. Based on the results, the abundances of transcripts and the genes expressed were calculated as read counts per sample.

### 2.10. Statistical Analysis

All statistical analyses were performed with SigmaStat statistical software (ver. 14.0-ESD, San Francisco, CA, USA). Continuous values for results of statistical analysis were expressed as the means ± SD. For comparisons between two groups, a Mann–Whitney U-test was used for continuous variables and a χ^2^ test for categorical variables. Overall survivals were calculated using the Kaplan–Meier method. Pearson’s correlation coefficient was used to measure the statistical relationship between two continuous variables. Cohen’s kappa coefficient was performed to evaluate the concordance between plasma, bile and FFPE. A difference with a *p* value of <0.05 was considered statistically significant.

## 3. Results

### 3.1. Patient Characteristics

**Figure 1:**
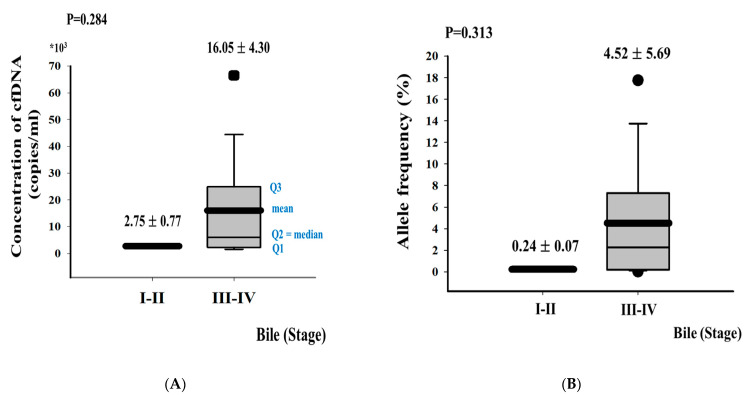

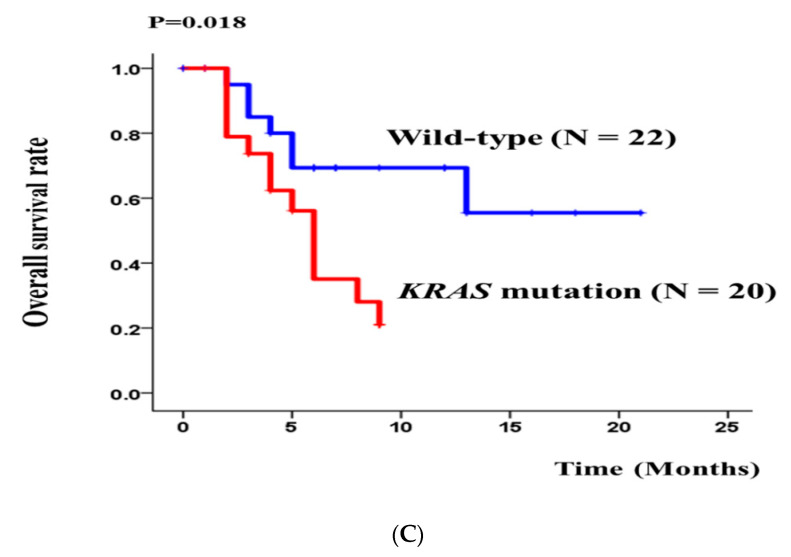
*KRAS* ctDNA concentrations and AF in bile according to clinical stages (**A**,**B**) and 2-year survival in patients with mutated or wild-type *KRAS* (**C**). Two-year survival was estimated with the Kaplan–Meier model.

The patient characteristics, including their age, sex, tumor site, method of obtaining bile, and cancer stage, are recorded in Table 1. Bile fluids were obtained from 46 patients with PTBD: 42 patients with BTC (BTC group) and 4 patients with a common bile duct (CBD) stones as the control. The patients with BTC included 23 men and 19 women, with a mean age of 73.2 ± 9.5 years (range 50–88 years). Among the patients with BTCs (N = 42), five had GBC, one had AC, and 36 had eCCA. Of these 42 patients, FFPE tumor tissue samples were available for 20 patients and frozen plasma samples, obtained preoperatively, were available for 16 patients. The clinical characteristics of the patients with wild-type and mutant *KRAS* are summarized according to sample type (bile, FFPE, and plasma) in Table 1. Sixteen patients underwent curative surgical resection and 26 patients received palliative chemotherapy or radiotherapy. The average concentration of bile ctDNA in advanced-stage (III/IV) BTCs was higher than in early-stage (I/II) BTCs, with significance (16,050 ± 18,249 vs. 2750 ± 777 copies/mL, respectively; *p* = 0.284; Figure 1A). Comparing to allele frequency (AF), the average of AF in advanced-stage (III/IV) BTCs was higher than in early-stage (I/II) BTCs (4.52 ± 5.69 vs. 0.24 ± 0.07 %, respectively; *p* = 0.313; Figure 1B); however, there were no statistical significance. In a Kaplan–Meier analysis, the patients with a *KRAS* mutation in bile ctDNA showed significantly worse survival than those with wild-type *KRAS* (2-year survival rates: 0% vs. 55.5%, respectively; *p* = 0.018) (Figure 1C). These results show that *KRAS* mutations in bile ctDNA can be used as a prognostic biomarker of BTCs.

### 3.2. DNA Fragment Size Distributions and Histograms of KRAS Mutations in Bile ctDNA, FFPE, and Plasma Samples

**Figure 2:**
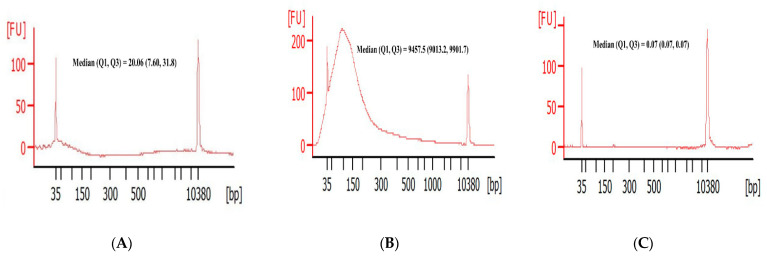

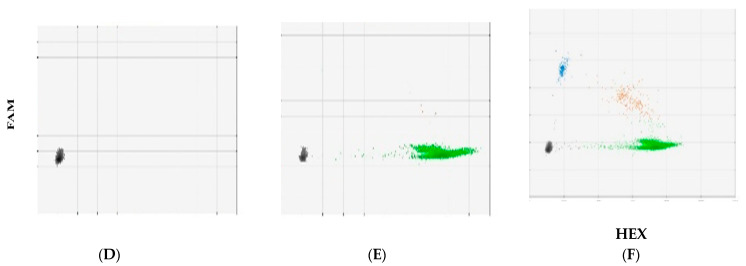
Size distribution of DNA fragments and histograms of *KRAS* mutations in bile (**A**), tissue (**B**) and plasma (**C**). Numbers indicate the sizes of the predominant DNAs in the samples. A 2D plots of the results of ddPCR *KRAS* multiplex assays applied to bile ctDNA in the NTC (**D**), control (**E**), and BTC samples with *KRAS* mutations (**F**). Black cluster on the plot represents negative droplets; green cluster represents droplets that were positive for wild-type DNA only; blue cluster represents droplets that were positive for mutant DNA only; and orange cluster represents droplets that were positive for both mutant and wild-type DNA. All panels show FAM amplitudes of up to 12,000 and HEX amplitudes of up to 12,000.

To observe the length of DNA fragments, we first compared the size distributions of the ctDNA molecules in the bile, plasma, and FFPE. The size of the DNA fragments was analyzed with an Agilent 2100 Bioanalyzer (Agilent Technologies, Inc.). As shown in Figure 2A, the majority of DNA fragments in bile were <100 bp, with a 10-bp periodicity of peaks observed (35~70 bp). Otherwise, The DNA fragments in FFPE samples (0~300 bp) were much larger than those in bile (Figure 2B). However, the maximum length was rarely seen in plasma (Figure 2C). To assess the mutant allele fraction, a *KRAS* duplex assay was applied to the bile, plasma, and tissue samples. Histograms of the NTC, control, and BTC mutations in bile are shown in Figure 2D–F. Histograms of the each mutant *KRAS* multiplex in bile, FFPE, plasma were displayed in Supplementary Figure S1. All the panels show a fluorescent amplitude molecule (FAM) amplitude of up to 12,000 and a highly expanded x-axis (HEX) amplitude of up to 12,000.

### 3.3. Concordance of KRAS Mutations among ctDNA in Bile, Plasma, and FFPE DNA Samples from BTC Patients

**Figure 3:**
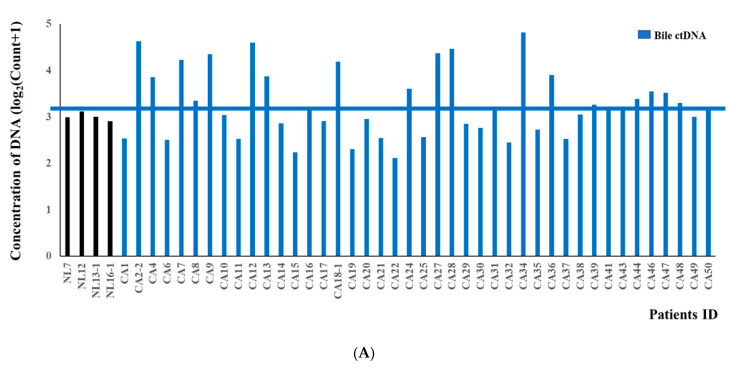

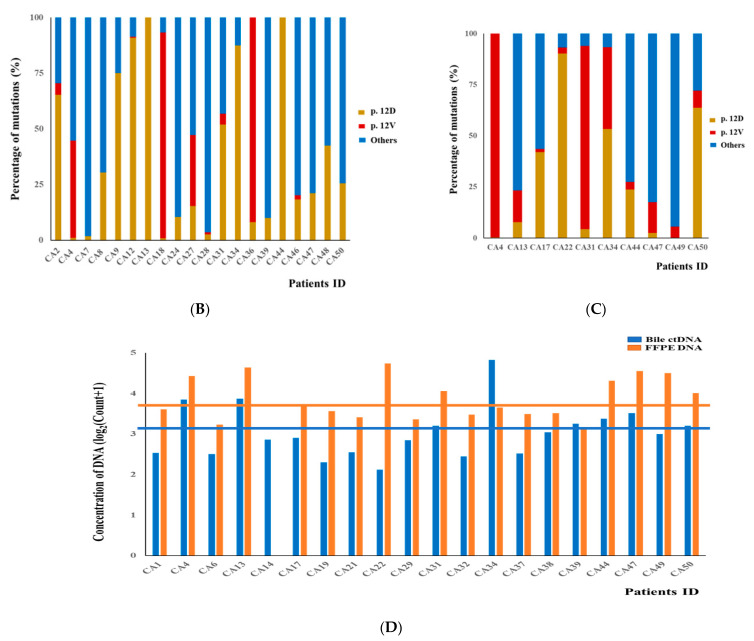
*KRAS* duplex assay and DNA concentrations in bile and FFPE samples. (**A**) *KRAS* duplex assay and ctDNA concentrations in all bile samples. Blue colored line indicates cut-off value for *KRAS* mutation in bile (1500 copies/mL). (**B**) Frequencies of G12D and G12V mutations in 20 patients with *KRAS* mutations in bile ctDNA. (**C**) Frequencies of G12D and G12V mutations in 10 patients with *KRAS* mutations in FFPE tissues. (**D**) Mutational concordance of *KRAS* between bile and paired FFPE samples (N = 20). Blue colored line indicates cut-off value for *KRAS* mutation in bile (1500 copies/mL) and orange colored line indicates that in FFPE (4500 copies/mL). The concentrations of mutant DNA (copies of mutant DNA per droplet) were estimated from the Poisson distribution. All panels show FAM amplitudes of up to 12,000 and HEX amplitudes of up to 12,000.

The concentrations of mutant *KRAS* DNA (copies of mutant DNA per droplet) were estimated from a Poisson distribution. The mean concentration of mutant DNA in the bile ctDNA from BTC patients (14,995 copies/mL) was about 10-fold higher than that of the control patient with a CBD stone (1000 copies/mL; Figure 3A), and we set the cut-off value for *KRAS* mutations at >1500 copies/mL (Table 2). All the *KRAS* mutations and *KRAS* allele frequencies from the original bile samples are listed in Supplementary Table S1. *KRAS* mutations were identified in the bile ctDNA from 20 (48%) of the BTC patients (N = 42) with a dual multiplex assay, and included G12D, G12V, G13D, G12A, G12C, G12R and G12S. *KRAS* allele frequencies were displayed in Supplementary Table S1. Among these 20 patients with *KRAS* mutations in their bile ctDNA, the frequencies of G12D and G12V were 20/20 (100%) and 9/20 (45%), respectively (Figure 3B).

In the FFPE samples, the mean concentration of FFPE DNA (24,515 copies/mL) was about 5-fold higher than in the control (4100 copies/mL). Therefore, we set the cut-off value for *KRAS* mutations at >4500 copies/mL (Table 2, Supplementary Table S2). Among 10 patients with *KRAS* mutation in FFPE, the majority of mutations were G12D or G12V (Figure 3C). We also compared the concentrations of bile ctDNA with the concentrations in the 20 paired samples of FFPE DNA to evaluate the mutational concordance. There was 80% mutational concordance between the paired bile ctDNA and FFPE samples with Cohen’s kappa coefficient of 0.600 (*p* = 0.006) (16/20) (Figure 3D). These results indicate that the changes in *KRAS* had significant prognostic implications and confirmed the mutational concordance between bile ctDNA and paired FFPE DNA in patients with BTCs.

However, the concentration of mutant *KRAS* ctDNA in plasma was very low and *KRAS* mutations and *KRAS* allele frequencies were only detected in three samples (18.8%), with a cut-off value of 60 copies/mL (Table 2, Supplementary Table S3). Among the 16 plasma samples, 14 samples were paired with FFPE samples and the mutational concordance rate was 42.9% (6/14) with Cohen’s kappa coefficient of 0.037 (*p* = 0.825).

### 3.4. Transcriptomic Sequencing in Bile, and Paired Plasma and Tissue

**Figure 4:**
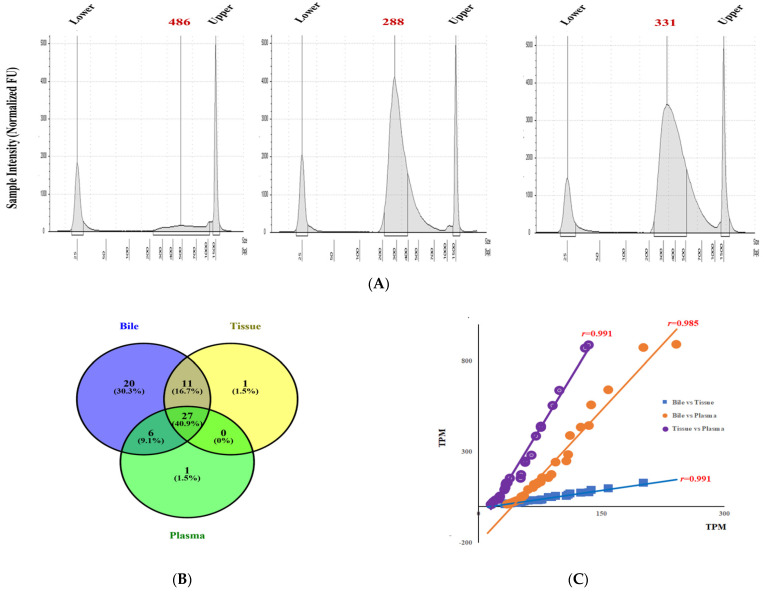
Transcriptomic sequencing of bile, tissue, and plasma samples. (**A**) Representative electropherograms for bile, paired tissue, and plasma mRNAs detected with an Agilent 2100 Bioanalyzer. (**B**) Venn diagram of the number of *KRAS*-associated mRNAs expressed in bile, paired tissue, and plasma samples from BTC patients, which were analyzed separately. (**C**) Scatter plot of the correlation matrix for expression of 66 *KRAS*-associated oncogenes evaluated in bile, paired tissue, and plasma samples (from patient CA34). Each dot represents the transcripts per million read (TPM).

In one patient for whom bile, plasma, and tissue samples were available, transcriptomic sequencing was performed to verify the overall mDNA expression levels in the bile, tissue and plasma. We checked the template size distribution by running the DNA samples on an Agilent Technologies 2100 Bioanalyzer using a DNA 1000 chip (RNA fragments >200 bp). Weakly expressed mRNAs, with <10 transcripts per million read, in the bile, tissue, and plasma were excluded from the analysis. The reference gene annotation for Homo Sapiens (GRCh38; release 109.20190607) was retrieved from the National Center for Biotechnology Information (NCBI). The profile of *KRAS* gene expression was determined with RNA-Seq by Expectation-Maximization (RSEM) (v1.3.1) with the options: estimate, rspd; seed-length, 15; strandedness, forward. Representative electropherograms for bile, the paired tissue, and plasma mRNAs are shown in Figure 4A. In total, we observed 46,425 genes and 66 *KRAS*-associated signaling oncogenes, including *RAS* and *RAF*, among the three groups (Figure 4B, Supplementary Table S4). The Venn diagram showing numbers of shared *KRAS* signaling oncogenes, and 57.6% of the *KRAS*-associated signaling oncogenes were expressed in both bile and tissue (Figure 4B). A correlation analysis indicated that the expression level of 66 *KRAS*-associated signaling oncogenes in the bile and tissue samples showed a strong positive correlation (*r =* 0.991, *p* < 0.001), as did those in the bile and plasma samples (*r* = 0.985, *p* < 0.001) and tissue and plasma (r = 0.991, *p* < 0.001) (Figure 4C). These results demonstrate that the *KRAS* oncogene expression detected with mRNA-Seq in bile reflects its expression in the tumor. Moreover, many oncogenes that are expressed in BTCs, such as *BRAF* and *NRAS*, were also detected in bile (data not shown). These data suggest that both gene expression and mutations can be detected in bile and that bile reflects the gene expression profile of the BTC tissue.

## 4. Discussion

Endoscopic or percutaneous tissue biopsy for the diagnosis of BTCs is invasive and its accuracy varies (50–90%) [12,29]. Therefore, tissue biopsy in BTC patients is challenging, and current guidelines do not recommend routine tissue biopsy for the diagnosis of BTC [30,31]. However, ctDNA, which is released into the bloodstream from dead tumor cells, has several advantages over tissue biopsy in precision medicine, including sampling convenience and dynamic monitoring. However, the proportion of ctDNA in the blood is extremely low, so extremely sensitive methods are required to detect mutations, for which the allelic frequencies can be as low as 0.1% [32]. In BTCs, bile fluid always directly contacts the tumor cells, and tumor-derived materials are abundant in the bile fluid. Moreover, BTCs contain heterogeneous features within a single mass, and the oncological information obtained from bile better reflects the molecular characteristics of the tumor than a tissue biopsy. Few studies have used bile ctDNA for the analysis or diagnosis of BTC. NGS has revealed that mutated tumor DNA can be detected in bile, and that the concordance rate with tumor tissue is high [16,33]. However, these studies included a small numbers of patients, and the clinical significance of the mutant DNA was unclear. Few studies have assessed the usefulness of bile fluid for the detection of ctDNA [34]. Moreover, NGS is expensive, so its clinical application is limited. ddPCR has many advantages over NGS in terms of shorter run-time, lower limits of detection, and greater cost effectiveness, together with its efficacy in determining the copy numbers of specific regions of DNA [35,36]. Most importantly, ddPCR can very accurately measure mutations that constitute only 0.01% of the samples. In the present study, we showed that shorter cell-free DNA fragments increased the proportion of ctDNA in bile. For the library in RNA-seq, an overlap process is not required. The reason is that the smaller the fragment size, the larger the overlap between reads. These results may provide compelling evidence that ctDNA fragment lengths described in our study may improve detection of non-metastatic solid tumors. Our findings may have a direct impact on the clinical utility of ctDNA for the non-invasive detection and diagnosis of solid tumors (i.e., the “liquid biopsy”), monitoring tumor recurrence, and evaluating tumor response to therapy.

In the present study, we have shown that liquid biopsy of bile, analyzed with ddPCR, can be used for the detection of DNA mutations. In addition, with transcriptomic sequencing, the expression of many genes encoding oncogenic signaling proteins, including *KRAS*, was detected at the same levels in the bile and paired tumor tissues from patients with BTCs. It means that bile accurately reflects the biological and oncological properties of the tumor tissue. Clinically, mutant *KRAS* transcripts were frequently detected in bile associated with advanced-stage (III/IV) tumors, and those patients had significantly worse survival rates than patients with wild-type *KRAS* transcripts. Shen and colleagues showed that although some configuration and effects are similar to our invention, only NGS is used and A146V, 136M and G12D are described among the *KRAS* mutation, and no description of the prediction of the prognosis of BTCs [37]. Therefore, our results suggest that the liquid biopsy of bile can be used instead of tissue biopsy for ctDNA analysis, and that *KRAS* mutations have a role as biomarkers of prognosis in BTC patients. Furthermore, ctDNA monitoring can provide a real-time insight into the response to treatment and may guide therapeutic adaptation in the future.

The genetic profiles of ctDNA in bile and plasma can be evaluated, as well as those in tumor tissue. However, the ctDNA detection rates in plasma were very low in the present study. In several previous studies, the ctDNA detection rates (20–75%) and the concordance between tissue and plasma results (25–79%) was not high [11,38]. In the present study, the detection rate of *KRAS* mutations was just 18.8% for plasma ctDNA compared with 48.0% for bile ctDNA. Moreover, the concordance between the *KRAS* mutations detected in bile and tissues (80.0%) was higher than the concordance between those in plasma and tissue (42.9%). Therefore, bile is a better biofluid than blood for the analysis of ctDNA in BTC patients, and in this study, we have demonstrated this superiority of bile biopsy in BTC patients for the first time. In the context of obstructive jaundice caused by BTCs, the exposure of the biliary epithelium and cancer cells to bile can be extensive, so the concentration of ctDNA derived from the tumor tissue can be high. However, we could not use fresh plasma in our comparison, and it is possible that the use of frozen plasma negatively affected our results. Because no other study has compared bile and plasma for the detection of ctDNA, a further comparative study of bile and plasma is required. In addition, there are a few mutant cases with close value to cut off values (supplementary Table S1). In these cases, it is difficult to define with certainty whether there is a mutation or not. For example, there was one patient who had positive in bile, but negative in FFPE. We could not validate their results with Sanger sequencing and it is another weak point.

This study had several limitations. Although it had the largest number of patients yet used to study bile ctDNA, the number of cases included was still insufficient for statistical validation. Because it was retrospective study and not all patients underwent tissue biopsy, we could get FFPE and plasma sample from some patients. Therefore, we must confirm our findings with a larger number of patients. Furthermore, the invasive procedure of endoscopic retrograde nasobiliary drainage via ERCP or PTBD is required to obtain bile fluid. However, in cases of obstructive jaundice caused by BTC or benign biliary stricture, biliary decompression is often required. Differentiating BTCs from benign biliary stricture is still clinically challenging, so liquid biopsy using bile may have clinical utility.

## 5. Conclusions

ctDNA is abundant in bile samples, and somatic variants of oncogenes in bile ctDNA can be detected with ddPCR. The high mutational concordance between bile and tumor tissue indicates that liquid biopsy to extract bile ctDNA can be used to detect *KRAS* variants for the diagnosis and prognosis of BTCs. The use of bile biopsy may lead to more rational, personalized, targeted therapeutic approaches to BTCs in the future.

## Figures and Tables

**Table 1:** Clinical characteristics of patients according to the bile, FFPE, and plasma samples analyzed.

	Bile (N = 42)			FFPE (N = 20)			Plasma (N = 16)		
	Wild Type (N = 22)	KRAS Mutant (N = 20)	*p*	Wild Type (N = 10)	KRAS Mutant (N = 10)	*p*	Wild Type (N = 13)	KRAS Mutant (N = 3)	*p*
Age (Mean, SD)	74.4 ± 9.6	71.9 ± 9.5	0.408	75.3 ± 8.3	65.7 ± 8.8	0.022	73.1 ± 8.3	74.3 ± 1.5	0.802
Sex (Male, %)	10 (45.5)	13 (65.0)	0.337	6 (60.0)	7 (70.0)	0.774	7 (53.8)	2 (66.7)	1.000
AJCC stage (I, II/III, IV)	5 (22.7)/ 17 (77.3)	3 (15.0)/ 17 (85.0)	0.808	3 (30.0)/ 7 (70.0)	5 (50.0)/ 5 (50.0)	0.478	7 (53.8)/ 6 (46.2)	0 (0.0)/ 3 (100.0)	0.238
Type of cancer			0.544			0.589			0.809
eCCA	19 (86.4)	17 (85.0)		9 (90.0))	8 (80.0)		12 (92.3)	3 (100.0)	
GBC	3 (13.6)	2 (10.0)		1 (10.0)	1(10.0)		0 (0.0)	0 (0.0)	
AC	0 (0.0)	1 (5.0)		0 (0.0)	1 (10.0)		1 (7.7)	0 (0.0)	
Treatment			0.476			1.000			0.809
Surgery	10 (45.5)	6 (30.0)		7 (70.0)	8 (80.0)		12 (92.3)	2 (66.7)	
Palliative care	12 (54.5)	14 (70.0)		3 (30.0)	2 (20.0)		1 (7.7)	1 (33.3)	

AJCC; American Joint Committee on Cancer, eCCA; extrahepatic cholangiocarcinoma, GBC; gallbladder cancer, AC; ampulla vater cancer.

**Table 2:** Incidence of *KRAS* mutations and concentrations of DNA in bile ctDNA, FFPE DNA, and plasma DNA from BTC patients. The 2D plotted data (black dots: FAM^−^/HEX^+^; blue dots: FAM^−^/HEX^+^; brown dots: FAM^−^/HEX^+^; green dots: FAM^−^/HEX^+^) from the ddPCR multiplex *KRAS* assay. Both the 12D and 12V point mutations were detected in the bile ctDNA, plasma DNA, and FFPE DNA samples. Presence of mutation was defined by copy number variation as cut-off value (bile > 1500 copies/mL, plasma > 60 copies/mL, and FFPE > 4500 copies/mL). All panels show FAM amplitudes of up to 12,000 and HEX amplitudes of up to 12000.

	Bile (N = 42)		FFPE (N = 20)		Plasma (N = 16)	
	N (%)	Concentration of DNA (Copies/mL)	N (%)	Concentration of DNA (Copies/mL)	N (%)	Concentration of DNA(Copies/mL)
***KRAS* multiplex mutation**	20 (47.6)	14,995.0 ± 17,602.6	10 (50.0)	24,515.0 ± 17,128.9	3 (18.7)	12.5 ± 26.9
***KRAS*** **point mutation**						
**p. G12D**	20 (100.0)	7917 ± 15,436.7	9 (90.0)	6982.0 ± 14,951.6	1 (33.3)	4.3 ± 17.5
**p. G12V**	9 (45.0)	1775.5 ± 3782.3	10 (100.0)	5647.0 ± 8185.9	3 (100.0)	11.8 ± 25.6
**p. G12D + p. G12V**	20 (100.0)	9692.5 ± 15,287.9	10 (100.0)	12,632.0 ± 15,207.5	3 (100.0)	16.2 ± 37.5

Concentration of *KRAS* DNA (copies/mL) was expressed as mean ± standard deviation.

## Data Availability

The original contributions presented in the study are included in the article, further inquiries can direct to the corresponding author.

## Supplementary Materials

The following are available online at www.mdpi.com/article/10.3390/cancers13184581/s1, Supplementary Figure S1: mutant KRAS multiplex in bile, FFPE, plasma, Supplementary Table S1: Concentration of mutant KRAS multiplex detected in bile, Table S2: Concentration of mutant KRAS multiplex detected in paired FFPE, Table S3: Concentration of mutant KRAS multiplex detected in paired plasma, Table S4: KRAS-associated signaling oncogenes, including RAS and RAF, with transcripts per million read (TPM) values for the bile, tissue, and plasma samples (‘0′ indicates that TPM > 10).
