# Supplementary material for: Liquid Biopsy from Bile-Circulating Tumor DNA in Patients with Biliary Tract Cancer
Source: Cancers (Basel). 2021 Sep 12;13(18):4581. doi: 10.3390/cancers13184581 (PMC8466375; doi:10.3390/cancers13184581)
Supplement: Supplementary file 1 [file cancers-13-04581-s001.zip › supplementary.pdf]

Table S1. Concentration of mutant *KRAS* multiplex detected in bile.

Patient ID	Concentration of DNA (copies/ml)	Copies/20ul	Positives	Negatives	Accepted Droplets	Allele frequency (%)
**NTC**	0	0	0	14959	14959	0
**NL7**	990	19.8	12	14286	14298	0.127
**NL12**	1300	26	17	15371	15388	0.611
**NL13-1**	1000	20	12	14773	14785	0.101
**NL16-1**	810	16.2	11	15897	15388	0.255
**CA1**	340	6.8	4	13805	13809	0.121
**CA2-2**	42000	840	434	11950	12384	5.495
**CA4**	7100	142	85	14020	14105	13.343
**CA6**	320	6.4	3	10922	10925	0.061
**CA7**	16800	336	206	14291	14497	2.173
**CA8**	2200	44	32	16840	16872	0.190
**CA9**	22400	448	243	12635	12878	4.319
**CA10**	1100	22	12	12558	12570	0.141
**CA11**	330	6.6	56	14053	14057	0.028
**CA12**	39400	788	417	12255	12672	13.150
**CA13**	7500	150	60	14146	14206	12.79
**CA14**	720	14.4	9	14611	14620	0.095
**CA15**	170	3.4	2	13824	13826	0.023
**CA16**	1400	28	17	13893	13910	0.153
**CA17**	800	16	9	12943	12952	0.101
**CA18-1**	15400	308	195	14792	14987	4.210
**CA19**	200	4	2	11973	11975	0.132
**CA20**	900	18	10	13637	13647	0.287
**CA21**	350	7	4	13308	13312	0.069
**CA22**	130	2.6	2	17715	17717	0.084
**CA24**	4000	80	53	15475	15528	0.340
**CA25**	360	7.2	4	13134	13138	0.156
**CA27**	23500	470	216	10712	10928	3.910
**CA28**	29000	580	366	14649	15015	2.416
**CA29**	700	14	8	13298	13306	0.060
**CA30**	580	11.6	6	12198	12204	0.054
**CA31**	1600	32	24	17194	17218	0.168
**CA32**	280	5.6	4	16713	16717	0.514
**CA34**	66500	1330	700	12036	12736	17.752
**CA35**	530	10.6	6	13207	13213	0.130
**CA36**	7900	158	114	10532	10576	0.436
**CA37**	330	6.6	5	17669	17674	0.215
**CA38**	1110	22.2	16	17004	17020	0.340
**CA39**	1800	36	25	16106	16131	0.602
**CA41**	1400	28	21	17197	17218	0.192
**CA43**	1400	28	19	15452	15471	0.125
**CA44**	2400	48	29	15802	15831	0.195
**CA46**	3500	70	40	13436	13436	0.305
**CA47**	3300	66	51	17943	117994	0.298
**CA48**	2000	40	29	17290	17319	0.169
**CA49**	1000	20	1	15864	17827	0.056

CA50	1600	32	24	17803	17827	0.143

Concentration of mutant *KRAS* p. G12D detected in bile

Patient ID	Concentration of DNA(copies/ml)	Copies/20ul	Positives	Negatives	Accepted Droplets
NTC	0	0	0	17232	17232
CA2-2	27400	0.548	349	14819	15168
CA4	70	1.4	1	16624	16625
CA7	320	6.4	5	18519	18524
CA8	670	13.4	10	17508	17518
CA9	16800	33.6	262	18243	18505
CA12	35800	71.6	554	17909	18463
CA13	7500	150	103	16086	16189
CA18-1	150	3	2	15210	15212
CA24	420	8.4	7	19505	19512
CA27	3600	72	53	17133	17186
CA28	760	15.2	10	15468	15478
CA31	830	16.6	12	16988	17000
CA34	58200	1164	730	14407	15137
CA36	640	12.8	9	16497	16506
CA39	180	3.6	3	19763	19766
CA44	2400	48	36	17352	17388
CA46	640	12.8	9	16668	16677
CA47	700	14	11	18498	16677
CA48	850	17	13	18075	18088
CA50	410	8.2	6	17240	17246

Concentration of mutant *KRAS* p. G12V detected in bile

Patient ID	Concentration of DNA(copies/ml)	Copies/20ul	Positives	Negatives	Accepted Droplets
NTC	0	0	0	15760	15760
CA2-2	2200	4.4	27	14654	14681
CA4	3100	6.2	47	17671	17718
CA7	0	0	0	17765	17765
CA8	0	0	0	17571	17571
CA9	0	0	0	18754	18754
CA12	190	3.8	3	18764	18767
CA13	0	0	0	14940	14940
CA18-1	14200	28.4	187	15392	15579
CA24	0	0	0	18841	18841
CA27	7500	1.5	97	15140	15237
CA28	270	0.54	4	17165	17169
CA31	80	1.6	1	15266	15267
CA34	0	0	0	8806	8806
CA36	7900	1.58	114	16908	17022

**CA39**	0	0	0	17305	17305
**CA44**	0	0	0	17898	17898
**CA46**	70	1.4	1	16782	16783
**CA47**	0	0	0	17831	17831
**CA48**	0	0	0	18274	18274
**CA50**	0	0	0	17941	17941

Table S2. Concentration of mutant *KRAS* multiplex detected in paired FFPE.

Patient ID	Concentration of DNA (copies/ml)	Copies/20ul	Positives	Negatives	Accepted Droplets	Allele frequency (%)
**NTC**	0	0	0	16060	16060	0
**NL2**	0	0	0	16528	16528	0
**NL3**	0	0	0	16701	16701	0
**NL4**	4100	82	5	16723	16726	0.460
**CA1**	4100	82	47	13609	13656	0.526
**CA4**	27000	456	273	13973	14246	14.035
**CA6**	1700	34	23	16015	16038	0.309
**CA13**	44200	884	538	14045	14583	7.094
**CA14**	0	0	0	17146	17146	0
**CA17**	5000	100	72	17038	17110	0.756
**CA19**	3700	74	48	15311	15359	0.438
**CA21**	2600	52	35	15762	15797	0.524
**CA22**	54400	1088	728	15383	16111	5.930
**CA29**	2300	46	31	15574	15605	0.450
**CA31**	11500	230	173	17552	17725	2.630
**CA32**	3000	60	43	16571	16614	0.359
**CA34**	4500	90	47	16512	16575	0.447
**CA37**	3100	62	47	17989	18036	0.306
**CA38**	3300	66	49	17639	17688	0.489
**CA39**	1500	30	25	19277	19302	0.361
**CA44**	20500	40	21	15545	15566	0.153
**CA47**	35800	70	38	15705	15743	0.266
**CA49**	32000	64	32	15216	15248	0.218
**CA50**	10250	20	12	17620	17632	0.205

Concentration of mutant *KRAS* p. G12D detected in paired FFPE.

Patient ID	Concentration of DNA(copies/ml)	Copies/20ul	Positives	Negatives	Accepted Droplets
**NTC**	0	0	0	15966	15966
**CA4**	70	1.4	1	16015	16016
**CA13**	3400	680	389	13259	13648
**CA17**	2100	42	26	14316	14342
**CA22**	49100	982	495	11619	12114
**CA31**	500	10	8	18647	18655
**CA34**	2400	48	33	16157	16190

**CA44**	4860	97.2	5	15422	15427
**CA47**	890	17.8	1	17876	17877
**CA49**	0	0	0	17903	17903
**CA50**	6530	13.6	8	18526	18534

Concentration of mutant *KRAS* p. G12V detected in paired FFPE.

Patient ID	Concentration of DNA(copies/ml)	Copies/20ul	Positives	Negatives	Accepted Droplets
**NTC**	0	0	0	18450	18450
**CA4**	27000	540	279	12039	12318
**CA13**	6900	138	71	12019	12090
**CA17**	80	1.6	1	15037	15038
**CA22**	1600	32	16	11645	11661
**CA31**	10300	206	16	12107	12213
**CA34**	1800	36	22	14489	14511
**CA44**	760	15.2	1	18154	18155
**CA47**	5380	107.6	6	16613	16619
**CA49**	1790	35.8	2	16402	16404
**CA50**	860	15.2	1	19023	19024

Table S3. Concentration of mutant *KRAS* multiplex detected in paired plasma.

Patient ID	Concentration of DNA (copies/ml)	Copies/20ul	Positives	Negatives	Accepted Droplets	Allele frequency (%)
**NTC**	0	0	0	16390	16390	0
**NL1**	0	0	0	17796	7796	0
**NL2**	0	0	0	16218	16218	0
**CA1**	0	0	0	18555	18555	0
**CA17**	0	0	0	20111	20111	0
**CA21**	60	1.2	0	19051	19052	0
**CA22**	0	0	0	18394	18394	0
**CA29**	0	0	0	15289	15289	0
**CA30**	70	1.4	1	19051	19052	7.692
**CA31**	0	0	0	15971	15971	0
**CA32**	0	0	0	17916	17916	0
**CA34**	0	0	0	18310	18310	0
**CA38**	0	0	0	16676	16676	0
**CA39**	0	0	0	19055	19055	0
**CA44**	0	0	0	17088	17088	0
**CA46**	0	0	0	18040	18040	0
**CA47**	0	0	0	16461	16461	0
**CA49**	70	1.4	1	16832	16833	1.030
**CA50**	0	0	0	16138	16138	0

Concentration of mutant *KRAS* p. G12D detected in paired plasma.

Patient ID	Concentration of DNA(copies/ml)	Copies/20ul	Positives	Negatives	Accepted Droplets
**NTC**	0	0	0	15286	15286
**CA21**	0	0	0	16386	16387
**CA30**	70	1.4	1	19337	19337
**CA49**	0	0	0	15286	15286

Concentration of mutant *KRAS* p. G12V detected in paired plasma.

Patient ID	Concentration of DNA(copies/ml)	Copies/20ul	Positives	Negatives	Accepted Droplets
**NTC**	0	0	0	13728	13728
**CA21**	60	1.2	1	20320	20321
**CA30**	60	1.2	1	19707	19708
**CA49**	70	1.4	1	16786	16787

Table S4. ***KRAS*-associated signaling oncogenes, including *RAS* and *RAF*, with transcripts per million read (TPM) values for the bile, tissue, and plasma samples ('0' indicates that TPM > 10).**

Gene_Symbol	Description	Bile_mRNA	Tissue_mRNA	Plasma_mRNA
MRAS	muscle RAS oncogene homolog	0	-	-
RAB10	RAB10, member RAS oncogene family	0	0	0
RAB11A	RAB11A, member RAS oncogene family	0	0	0
RAB11B	RAB11B, member RAS oncogene family	0	0	0
RAB12	RAB12, member RAS oncogene family		0	-
RAB13	RAB13, member RAS oncogene family	0	0	0
RAB14	RAB14, member RAS oncogene family	0	0	0
RAB15	RAB15, member RAS oncogene family	0	-	-
RAB17	RAB17, member RAS oncogene family	0	0	-
RAB18	RAB18, member RAS oncogene family	0	0	0
RAB1A	RAB1A, member RAS oncogene family	0	0	0
RAB1B	RAB1B, member RAS oncogene family	0	0	-
RAB20	RAB20, member RAS oncogene family	0	0	-
RAB21	RAB21, member RAS oncogene family	0	0	0
RAB22A	RAB22A, member RAS oncogene family	0	0	-
RAB24	RAB24, member RAS oncogene family	0	0	0
RAB25	RAB25, member RAS oncogene family	0	0	-
RAB26	RAB26, member RAS oncogene family	0	-	-
RAB27A	RAB27A, member RAS oncogene family	0	0	0
RAB27B	RAB27B, member RAS oncogene family	0	0	0
RAB28	RAB28, member RAS oncogene family	0	0	-
RAB29	RAB29, member RAS oncogene family	0	-	-
RAB2A	RAB2A, member RAS oncogene family	0	0	0
RAB2B	RAB2B, member RAS oncogene family	0	0	-
RAB30	RAB30, member RAS oncogene family	0	-	0
RAB31	RAB31, member RAS oncogene family	0	0	0
RAB32	RAB32, member RAS oncogene family	0	-	0
RAB33A	RAB33A, member RAS oncogene family	0	-	0
RAB33B	RAB33B, member RAS oncogene family	0	0	-
RAB34	RAB34, member RAS oncogene family	0	0	-
RAB35	RAB35, member RAS oncogene family	0	0	0
RAB36	RAB36, member RAS oncogene family	0	-	-
RAB37	RAB37, member RAS oncogene family	0	-	-
RAB38	RAB38, member RAS oncogene family	0	-	0
RAB39A	RAB39A, member RAS oncogene family	0	-	-
RAB3A	RAB3A, member RAS oncogene family	0	-	0
RAB3B	RAB3B, member RAS oncogene family	0	-	-
RAB3D	RAB3D, member RAS oncogene family	0	0	-
RAB40A	RAB40A, member RAS oncogene family	0	-	-
RAB40B	RAB40B, member RAS oncogene family	0	-	-
RAB40C	RAB40C, member RAS oncogene family	0	-	-
RAB42	RAB42, member RAS oncogene family	0	-	-
RAB42P1	RAB42, member RAS oncogene family, pseudogene 1	0	-	-
RAB43	RAB43, member RAS oncogene family	0	-	-
RAB44	RAB44, member RAS oncogene family	0	-	-
RAB4A	RAB4A, member RAS oncogene family	0	0	0
RAB4B	RAB4B, member RAS oncogene family		-	0
RAB5A	RAB5A, member RAS oncogene family	0	0	0
RAB5B	RAB5B, member RAS oncogene family	0	0	0
RAB5C	RAB5C, member RAS oncogene family	0	0	0
RAB6A	RAB6A, member RAS oncogene family	0	0	0
RAB6B	RAB6B, member RAS oncogene family	0	-	0
RAB6C	RAB6C, member RAS oncogene family	0	-	-
RAB7A	RAB7A, member RAS oncogene family	0	0	0
RAB8A	RAB8A, member RAS oncogene family	0	0	0
RAB8B	RAB8B, member RAS oncogene family	0	0	0
RABL2A	RAB, member of RAS oncogene family like 2A	0	-	-
RABL2B	RAB, member of RAS oncogene family like 2B	0	-	-
RABL3	RAB, member of RAS oncogene family like 3	0	-	-
RABL6	RAB, member RAS oncogene family like 6	0	0	0
RAN	RAN, member RAS oncogene family	0	0	0
RAP1A	RAP1A, member of RAS oncogene family	0	0	0
RAP1B	RAP1B, member of RAS oncogene family	0	-	0
RAP2A	RAP2A, member of RAS oncogene family	0	0	0
RAP2B	RAP2B, member of RAS oncogene family	0	0	0
RAP2C	RAP2C, member of RAS oncogene family	0	0	-

Figure S1. mutant *KRAS* multiplex in bile, FFPE, plasma

<Bile>

- mutant *KRAS* multiplex detected in CBD bile

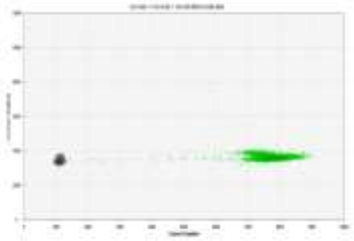

<NL7>

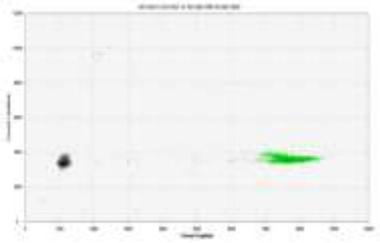

<NL12>

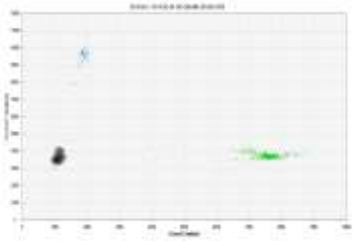

<NL13-1>

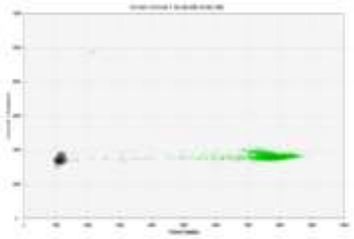

<NL16-1>

- mutant *KRAS* multiplex detected in BTC bile.

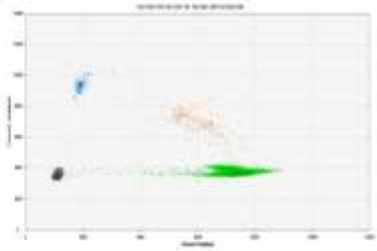

<CA2-2>

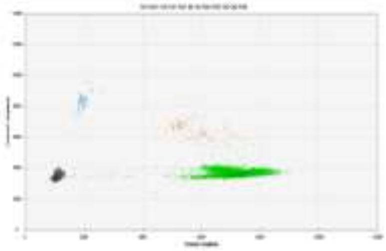

<CA7>

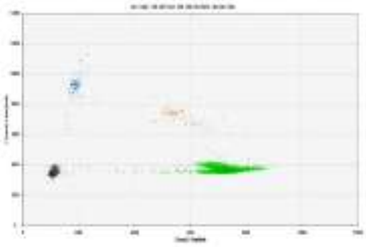

<CA9>

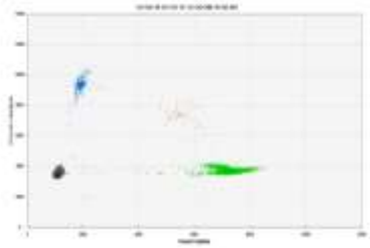

<CA12>

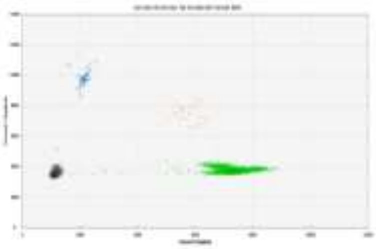

<CA18-1>

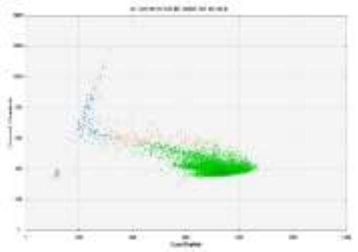

<CA27>

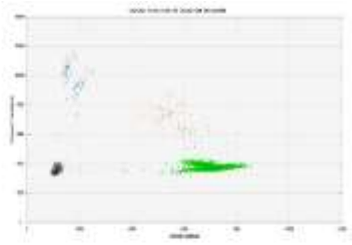

<CA28>

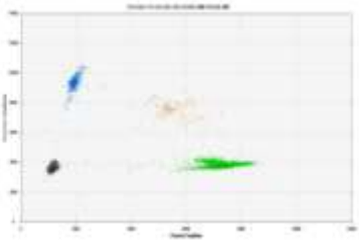

<CA34>

<FFPE>

- mutant *KRAS* multiplex detected in non-tumor area of FFPE.

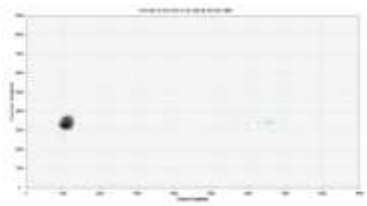

<NL2>

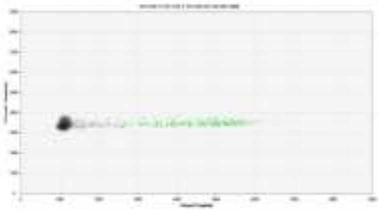

<NL3>

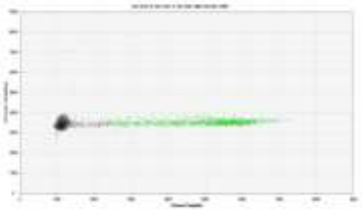

<NL4>

- mutant *KRAS* multiplex detected in BTC FFPE.

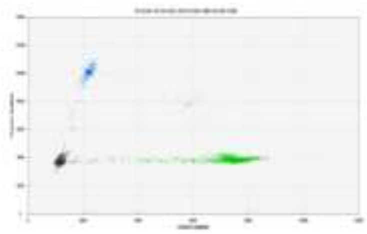

<CA4>

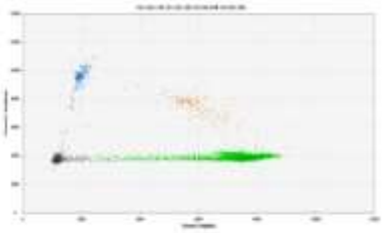

<CA13>

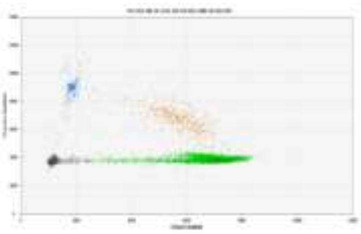

<CA22>

<Plasma>

mutant *KRAS* multiplex detected in control plasma.

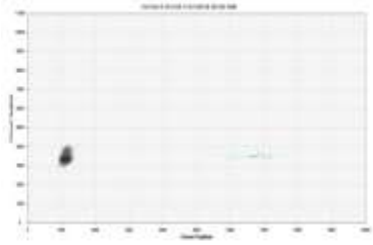

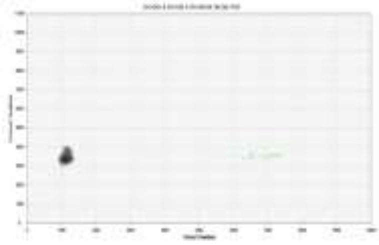

<NL1> <NL2>

- mutant *KRAS* multiplex detected in BTC plasma.

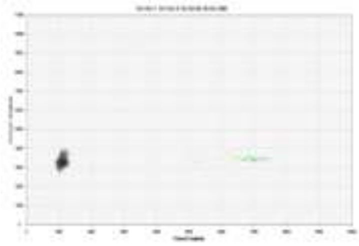

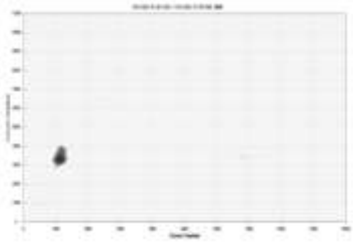

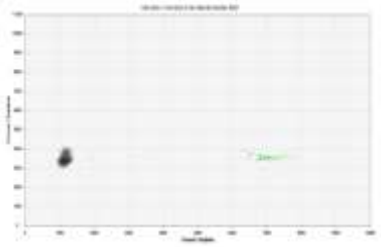

<CA21> <CA30> <CA49>
